# Enhancing the study on antithrombotic therapy after stroke in AF patients

**DOI:** 10.1093/europace/euae147

**Published:** 2024-06-05

**Authors:** Shen Wang

**Affiliations:** School of Public Health, China Medical University, 77 Puhe Road, Shenbei New District, Shenyang, Liaoning Province 110122, China; School of Medicine, Dentistry and Biomedical Sciences, Queen's University Belfast, Whitla Medical Building, 97 Lisburn Road, Belfast, Northern Ireland BT9 7BL, UK

We read with great interest the research paper entitled ‘Association between antithrombotic therapy after stroke in patients with atrial fibrillation and the risk of net clinical outcome: an observational cohort study’ by Ahn *et al.*^1^ The study provides valuable insights into the prescription patterns and long-term outcomes of antithrombotic therapy in patients with atrial fibrillation (AF) and acute ischaemic stroke (IS). The authors’ thorough investigation into these patterns and outcomes is commendable and contributes significantly to our understanding of secondary stroke prevention. However, we would like to address several points that we believe could enhance the study's robustness and applicability.

1. Definition of Bleeding Criteria in Primary Outcomes
  The study defines the net clinical outcome (NCO) by including any bleeding, which may introduce variability in the severity of bleeding events. Many studies define bleeding criteria more stringently, often focusing on major bleeding events as these have more significant clinical implications (e.g. ‘fatal bleedings’,^2^ ‘life-threatening bleeding’,^3^ ‘major bleeding’^4^). We suggest considering the use of major bleeding as a criterion for defining NCO, as this would provide a clearer and more clinically relevant measure of bleeding risks. What are the authors’ perspectives on this adjustment, and how might it impact the study's conclusions?
2. Inclusion of Chronic Kidney Disease as a Clinical Factor
  The study by Ahn *et al.* did not consider chronic kidney disease (CKD) as a factor in their analysis of bleeding risks associated with oral anticoagulants (OACs). According to Yamato *et al.*^5^, CKD is significantly associated with an increased risk of bleeding in patients on OACs.^5^ Given the substantial impact of CKD on bleeding risks, it would be beneficial to include CKD as a variable in future analyses to provide a more comprehensive understanding of the factors influencing bleeding risks in patients with AF and IS.
3. Reliability of ICD-10 Codes for Patient Identification
  To improve the reliability of patient identification, we suggest conducting sampling validation to assess the accuracy of the ICD-10 codes. Additionally, considering the integration of natural language processing techniques, as demonstrated by Johnson *et al.*^6^, could help extract more precise diagnostic information from electronic medical records. This approach would likely enhance the accuracy of patient identification and classification.
4. Addressing Selection Bias in Single-Centre Retrospective Studies
  The authors acknowledge the limitation of potential selection bias due to the study being conducted at a single tertiary hospital, which may not be representative of the broader population. To mitigate this bias and improve generalizability, we recommend expanding the study to multiple centres. Multi-centre studies can provide a more diverse patient population, reducing selection bias, and enhancing the external validity of the findings.^7^ We are interested in understanding the feasibility and potential challenges of expanding this research to include multiple centres.

We appreciate the contributions of Ahn *et al.* to the field of antithrombotic therapy and stroke prevention. Addressing the above points could further strengthen the study's impact and provide more actionable insights for clinical practice.
